# Supplementary material for: Abstract spatial, but not body-related, visual information guides bimanual coordination
Source: Sci Rep. 2017 Dec 1;7:16732. doi: 10.1038/s41598-017-16860-x (PMC5711801; doi:10.1038/s41598-017-16860-x)
Supplement: Supplementary file 1 — Supplemental Material [file 41598_2017_16860_MOESM1_ESM.pdf]

Supplemental Material

Abstract spatial, but not body-related, visual information guides bimanual coordination

Janina Brandes, Farhad Rezvani, & Tobias Heed

In the supplemental material, we address the following issues:

1. We demonstrate that sine fitting adequately represents the raw data.
2. We demonstrate that dichotomizing phase differences according to a +/-50° criterion leads to results that are qualitatively comparable to those obtained when using other analysis methods.
3. We demonstrate that the reported results do not mainly reflect a switch of movement from horizontal (instructed) to vertical (uninstructed) movement directions, but instead true reflect a reduction of (overall) movement accuracy.

1. Sine waves capture the velocity pattern of the raw data

A movement cycle's average phase information can be readily retrieved from a sine fit of the finger movement data. Movement velocity in the present task usually follows a sinusoidal velocity pattern, which is made explicit with this analysis approach. The main advantage of sine fitting is that it renders one single phase difference value that represents a movement cycle, rather than relying on identification of high and low points, which are sometimes ambiguous due to imperfect movement. However, the fitting implicitly introduces smoothing, and implicitly averages (by optimizing the fit) the phase differences of individual data points. Fig. S1 presents comparisons of raw data and sine fits of several randomly chosen trials; note, that raw and fitted data reveal very similar high and low points, indicating that the extracted phase values will be similar as well.

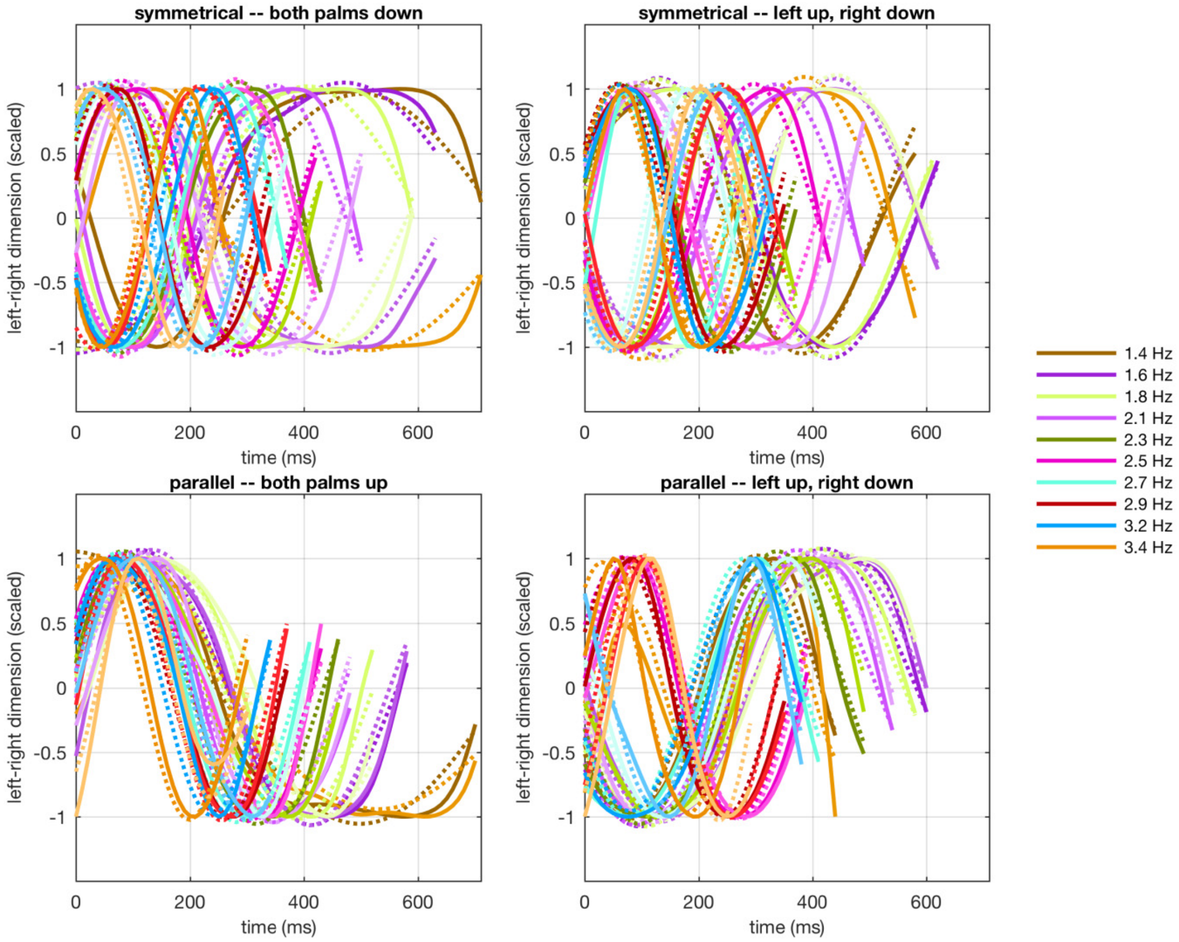

Figure S1: Illustration of exemplar sine wave fit to raw data of a single participant. One movement cycle is plotted per speed level (color-coded) and hand orientation across movement instructions (panels). Solid lines indicate raw data, whereas fitted sine waves are plotted as dashed lines. Dark and bright shades of the same color indicate left and right hand traces respectively.

However, to explicitly validate our analysis approach, we conducted two new analyses, both of which do not rely on sine fits. Both analyses render equivalent results to the ones we report in the main text.

In the first analysis, we extracted the relative phase of the two hands at each sampled time point during the movement cycle. To this end, we estimated the continuous phase of each hand using Discrete Fourier Transform (DFT) and Maximum Likelihood Estimation (MLE)[1]. This DFT/MLE approach has been suggested to be superior to other phase extraction approaches[2]. Next, we calculated the median difference between the phases of the two hands. Because cutting our multi-second trials into movement cycles lead to high variability of estimated phase at the edges of our cycle time courses, we excluded 15% of data points on each trial edge. For comparison with the results we report in the main paper, we analyzed the extracted phase differences in exactly the same way as we analyzed phase differences obtained via sine fitting. The results are shown in Figures S2-S5. Note that the pattern of results

closely resembles our original, sinefit-based result (main text; Figures 1- 3, 5).

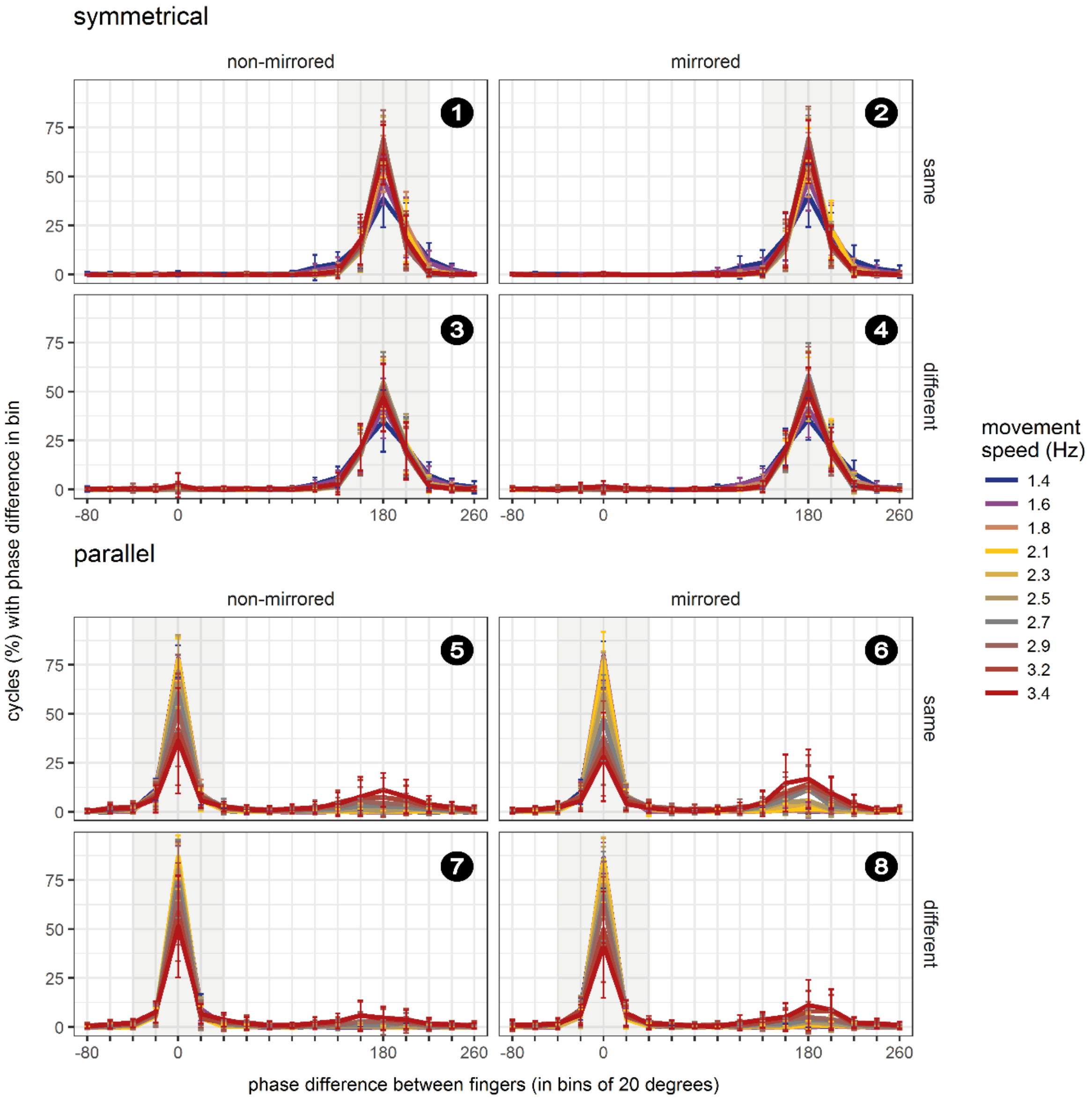

Figure S2: Performance in the finger oscillation task estimated without sinusoidal fitting. The figure was otherwise produced exactly like the one in our main text and can therefore be directly compared with it.

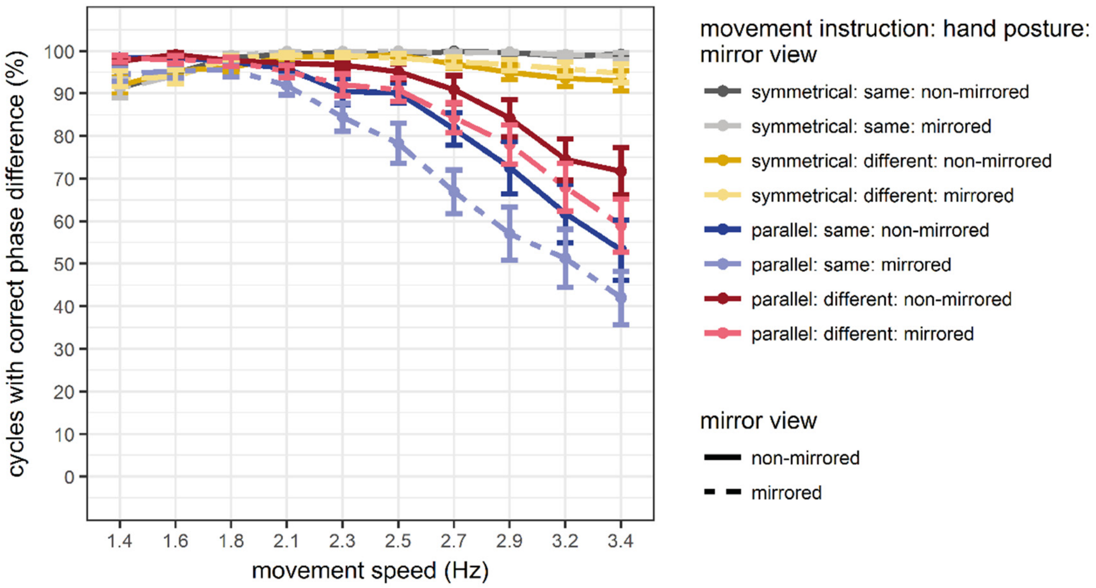

Figure S3: Accuracy in the finger oscillation task estimated without sinusoidal fitting. The figure was otherwise produced exactly like the figure in the main text and can, thus, be directly compared to it.

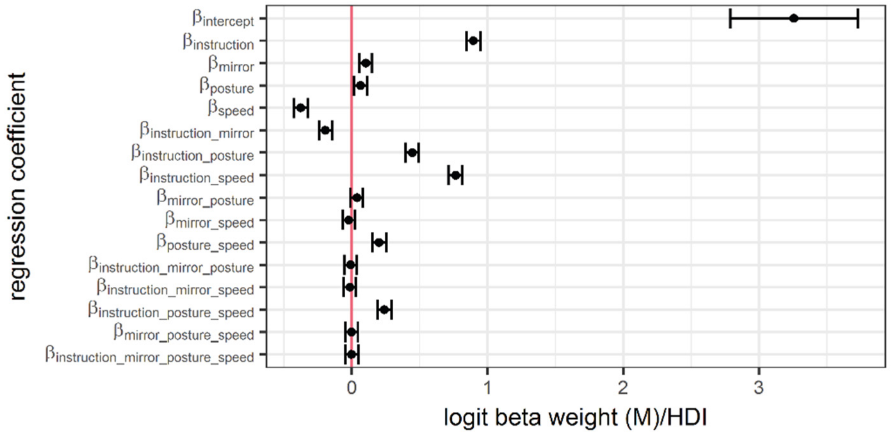

Figure S4: Illustration of the results of the statistical analysis as estimated without relying on sinusoidal fitting. The statistical procedure and figure plotting were identical to those of our original analysis in the main text; the figures can, thus, be directly compared.

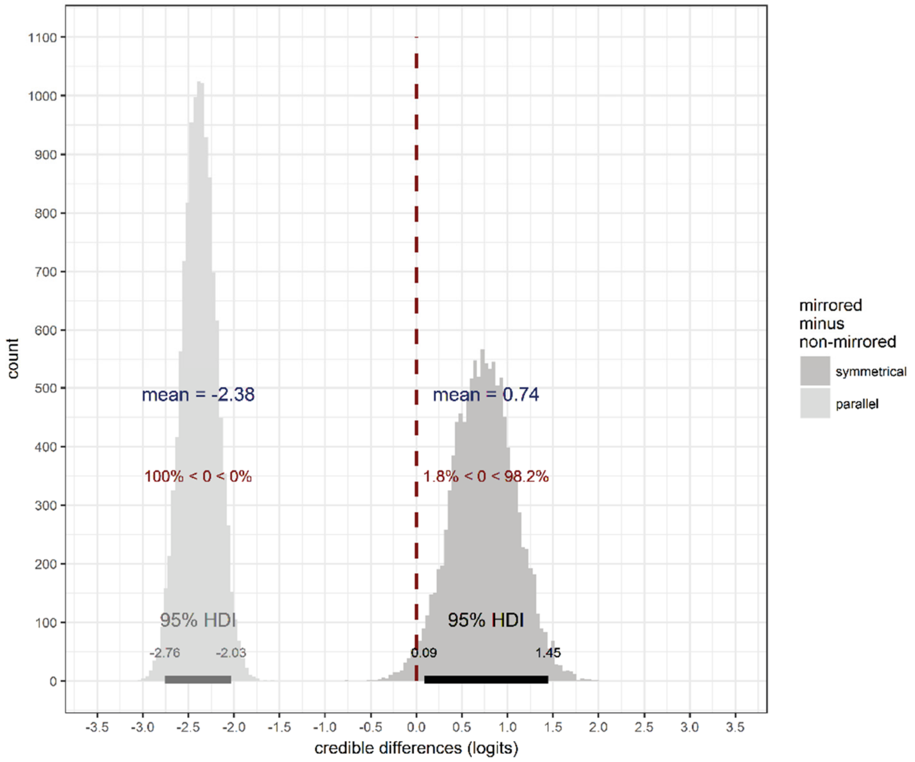

Figure S5: Illustration of credible difference distributions of the parameter $\beta_{instruction\_mirror}$ estimated within a Bayesian hierarchical logistic regression model run on the data obtained without sinusoidal fitting. The credible differences of the parameter $\beta_{instruction\_mirror}$ are shown, because the HDI representing the interaction parameter $\beta_{instruction\_mirror\_speed}$ covers zero (see Figure S3). The figure was otherwise created in the same way as that in the original analysis shown in the main paper and can, thus, be directly compared.

In a second additional analysis, we visualized the data using an alternative measure that has been suggested by Wilson and colleagues[3] using their kindly provided MATLAB code. This analysis aimed to further show that the effects on coordination stability we report do not depend on which measure is calculated, and how. In this analysis, one obtains a measure for the proportion of time in a target frequency. This measure is based on the entire trial segments for a given speed level, rather than on movement cycles. Thus, here we obtained a single dependent value per speed and trial that represents all respective movement cycles. This analysis approach avoids dichotomization of the data into “correct” or “incorrect” per movement cycle, as performed in the main text; instead, the whether the requested phase difference is produced by the participant is checked on each pair of sample points for the two fingers, similar to the approach we used in the above first additional analysis. Results are illustrated in Figure S6, which shows the same result pattern as obtained when calculating the “relative phase angle” with and without relying on sinusoidal fitting (see Figure 2 in the main text and Figure S3).

In sum, then, these additional analyses demonstrate that the results we report are qualitatively independent of the specific analysis method used, and incidentally validates these different approaches as largely equivalent in terms of their applicability for the type of paradigm we

employed.

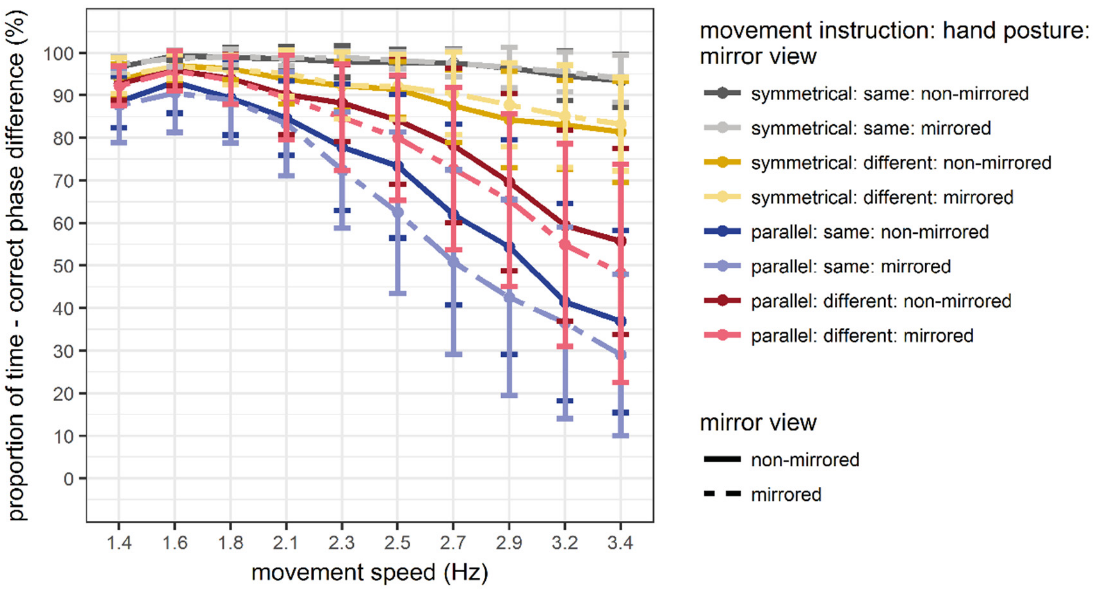

Figure S6: Accuracy in the finger oscillation task as estimated with the measure "proportion of time in target frequency". Error bars indicate the standard deviation (s.d.) of averaged proportions per speed level across participants. Apart from the different measure of variance shown (here s.d. instead of s.e.), the figure can be directly compared to our original figure (and to that of our first additional analysis, Figure S3).

2. *The reported results are qualitatively similar when phase differences are dichotomized according to different criteria*

By using a phase range of +/- 50° to determine of movements were performed correctly, we adopted an analysis strategy that has been introduced by others[4] and that we have used previously in the finger oscillation task[5]. Using this strategy also in the present study allows comparison with these previous studies.

Furthermore, the histograms displayed in Figure 1 in the manuscript illustrate that a +/- 50° relative phase range (grey shading) is appropriate to capture the major part of the distributions that center around the correct phase difference at slow movement speeds (180° for symmetrical and 0° for parallel movements). Choosing a smaller relative phase range cuts off large proportions of these distributions that describe the variation of relative phase at higher movement speeds in the present experiment. Nonetheless, to demonstrate that our central results are invariant to the threshold used to dichotomize the data into correct and false responses, Figure S7 shows accuracy using a relative phase range of +/-20° in correspondence to Figure 2 from the manuscript. Note, that the results are similar for both analyses (cut-off at 50° and at 20°).

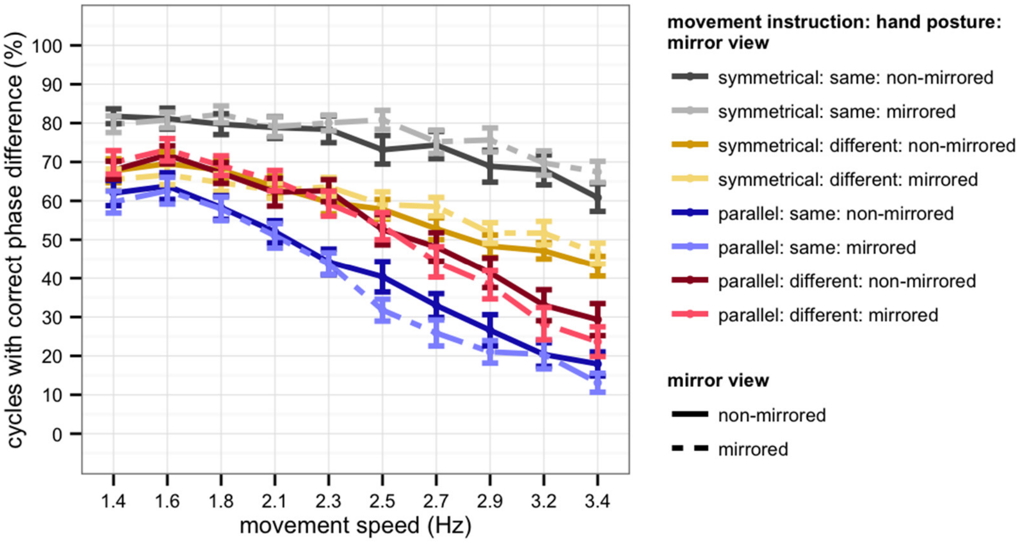

Figure S7: Illustration of accuracy in the finger oscillation task. Percentage of movement cycles with the correct phase difference (+/-20°) between the two index fingers. Line colors represent the interaction of movement instruction (symmetrical vs. parallel) and hand orientation (same vs. different). Dark colors and solid lines represent non-mirrored conditions, and bright colors and dashed lines indicate mirrored feedback conditions. Error bars represent standard errors of the mean.

To show that also the statistical pattern of results is independent of the threshold used to dichotomize the data, we re-estimated the Bayesian model using a threshold of 20°. Results of this analysis are shown in Figures S8 and S9. Note that the results closely resemble the original pattern of results, which is based on a 50° threshold (see Figures 3 and 5 in the main text).

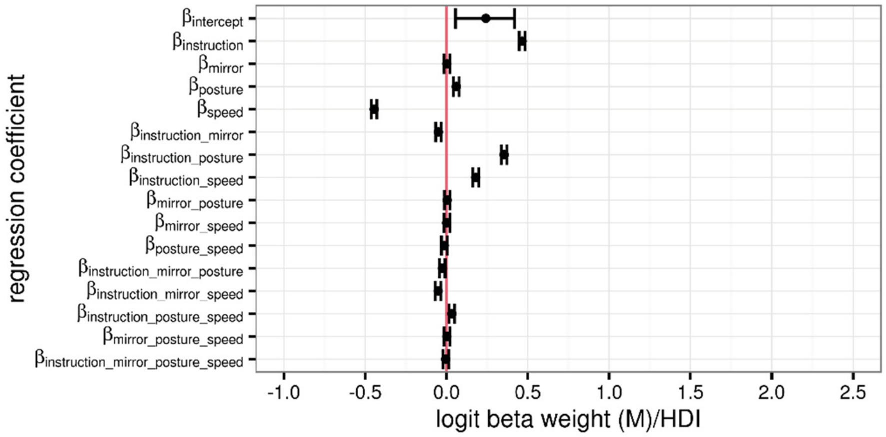

Figure S8: Illustration of the results of the statistical analysis as estimated with a threshold of 20°. The statistical procedure and figure plotting were identical to those of our original analysis in the main text; the figures can, thus, be directly compared.

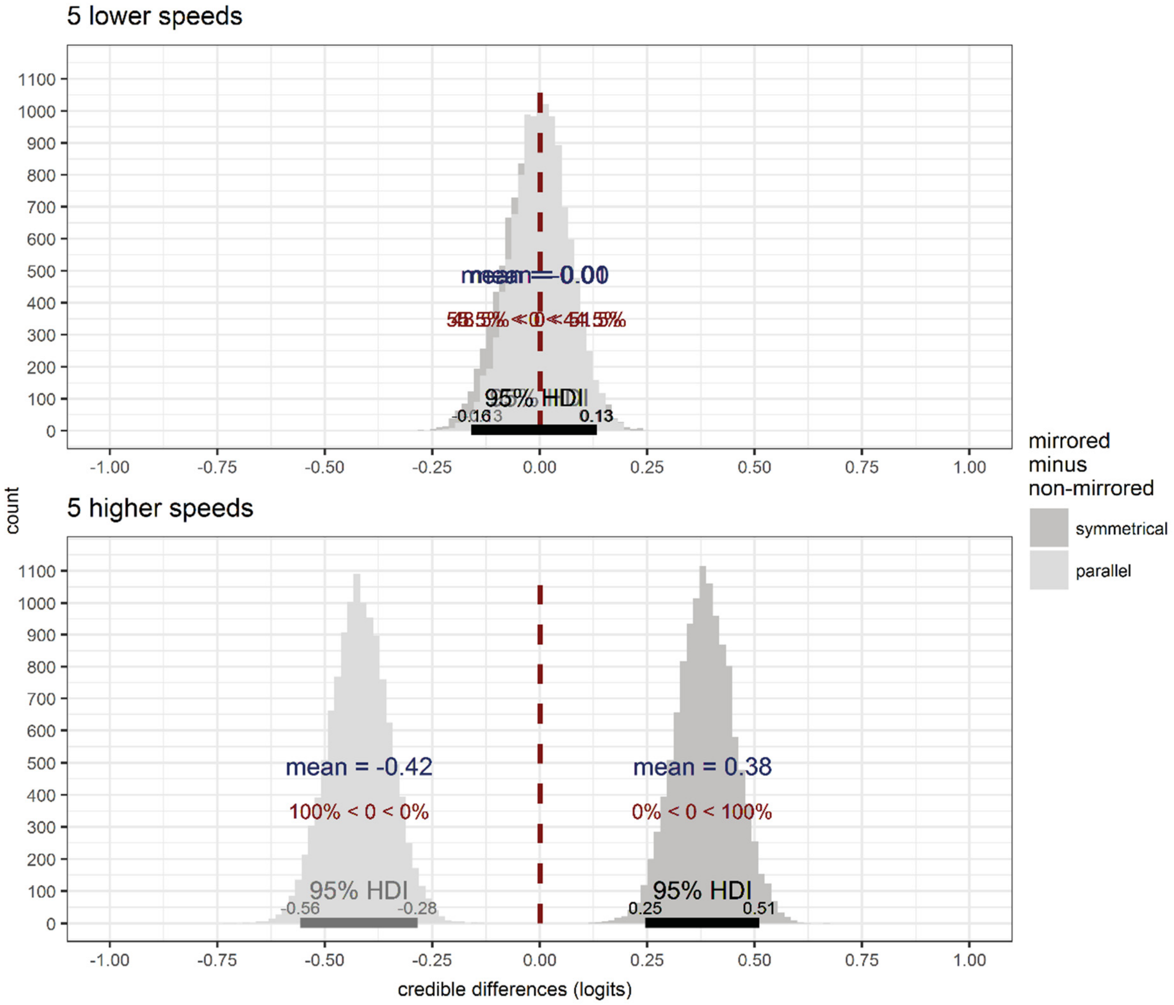

Figure S9: Illustration of credible difference distributions of the parameter $\beta_{instruction\_mirror\_speed}$ estimated within the Bayesian hierarchical logistic regression model using a threshold of 20°. The figure was otherwise created in the same way as that in the original analysis shown in the main paper and can, thus, be directly compared.

3. There is no trade-off between movements in the horizontal and vertical planes

Participants were required to perform abduction and adduction movements in the horizontal plane. Previous studies have shown that the motor system can spontaneously recruit additional degrees of freedom when high movement speed is required. For finger oscillations, motion can transition from the horizontal (left-right) toward the vertical (up-down) dimension. We note, that if such trade-offs were evident in our study, they would still reflect condition-specific modulations, and, thus, lead us to draw to similar conclusions as the ones we draw in the main text. Nevertheless, we conducted several analyses to test whether participants produced the instructed abduction and adduction movements in the horizontal plane at high speeds in the present study, and whether the effects we report reflect a trade-off between movements in the horizontal and vertical planes.

We analyzed movement velocity, because this variable best reflects the variability of the finger movements over time and is also the basis of the sine waves analyzed in the present study. First, Figure S10 illustrates density distributions fitted to the absolute number of movement cycles identified per participant at the four highest speed levels (ranging from 2.5. to 3.4 Hz), pooled across movement instructions and hand orientations. At high speeds, participants tend to get out of rhythm and reduce movement amplitude, and this often leads to a moderate decline of the number of performed movement cycles that can be identified in motion data. For simplicity, data are collapsed across mirror view conditions. The grand mean of movement cycles across participants is displayed as black horizontal bars; the instructed number of movement cycles is displayed as a red dashed line. It is evident from the shape of the density distributions that the number of produced movement cycles generally matched the target number of movements quite well for all participants, even in the most difficult condition, that is, parallel movements at high speeds with hands oriented differently (bottom right panel).

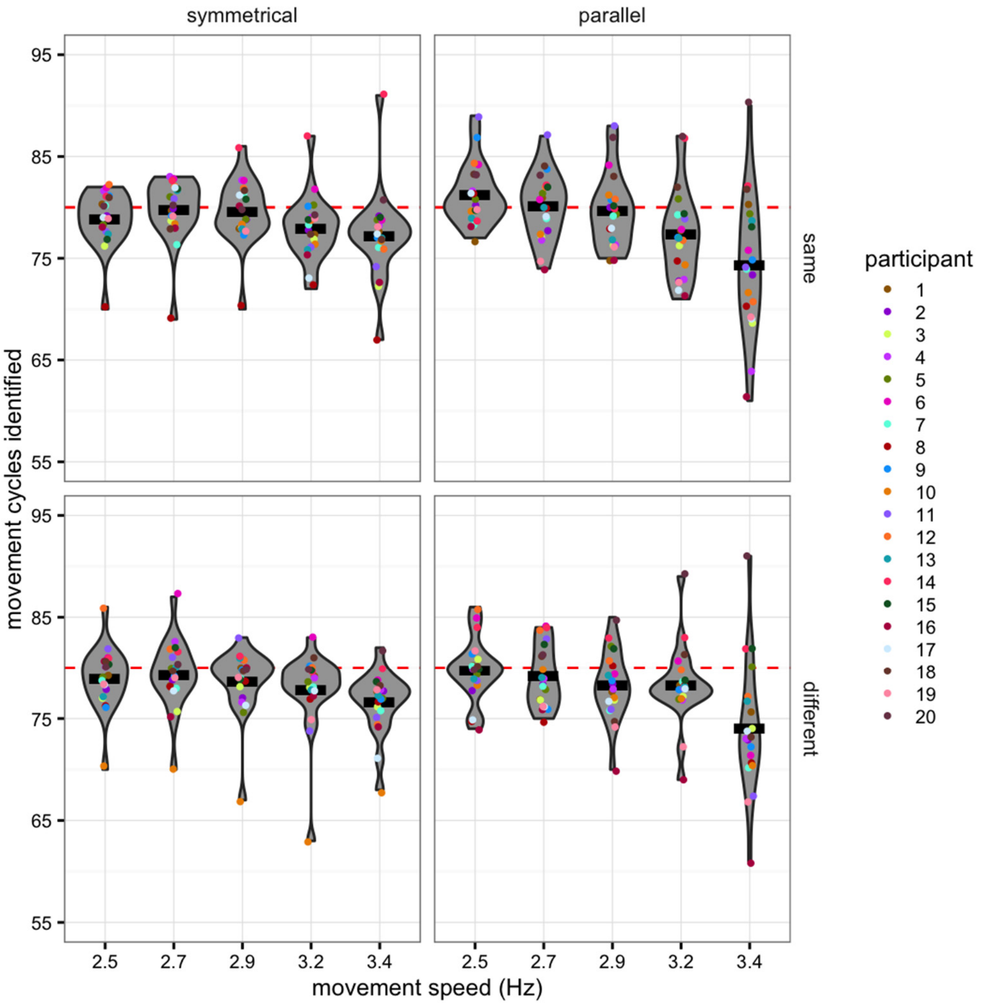

Figure S10: Illustration of the absolute number of movement cycles identified per participant at high speed levels (2.5 to 3.4 Hz).

Second, to demonstrate that movements were primarily executed in the horizontal plane at all times, Figure S11 shows numerically differentiated movement velocities of a single

participants’ left (blue) and right hand (red) in the left-right (horizontal) dimension across all hand orientations. Example data is taken from the most difficult condition, that is, parallel movements executed with mirrored visual feedback. If one presumes that systematic trade-offs occur between horizontal and vertical movements due to our experimental conditions, a switch to the vertical motion plane should be especially evident when performance is most difficult. Figure S12 shows the corresponding velocities in the up-down (vertical) dimension. It is apparent that amplitudes were highest in the left-right dimension, even at high frequencies and when executing parallel movements with hands oriented differently (two bottom panels), as well as in a supine orientation (two top panels).

Third, to illustrate this finding on a group level, Figure S13 shows the standard deviation of velocity per participant and speed level (ranging from 1.4 to 3.4 Hz) for all three movement dimensions, averaged across trials and the two hands. Standard deviations should be highest in the instructed movement plane, that is, the left-right dimension, while standard deviations in the other dimensions should be considerably smaller. For simplicity, data are collapsed across mirror view conditions. The grand mean of standard deviations across participants is displayed as horizontal bars that are color-coded according to movement dimension. Critically, the standard deviation of velocity increases in all three dimensions with rising movement speed. This result pattern does not support the suggestion that a trade-off from one to the other dimension has taken place, but instead reflects a general increase in movement variablilty with higher speed. Furthermore, amplitude in the horizontal dimension exceeded that of the other two dimensions even at high speeds in all participants. This finding holds also when considering each hand separately (data not shown).

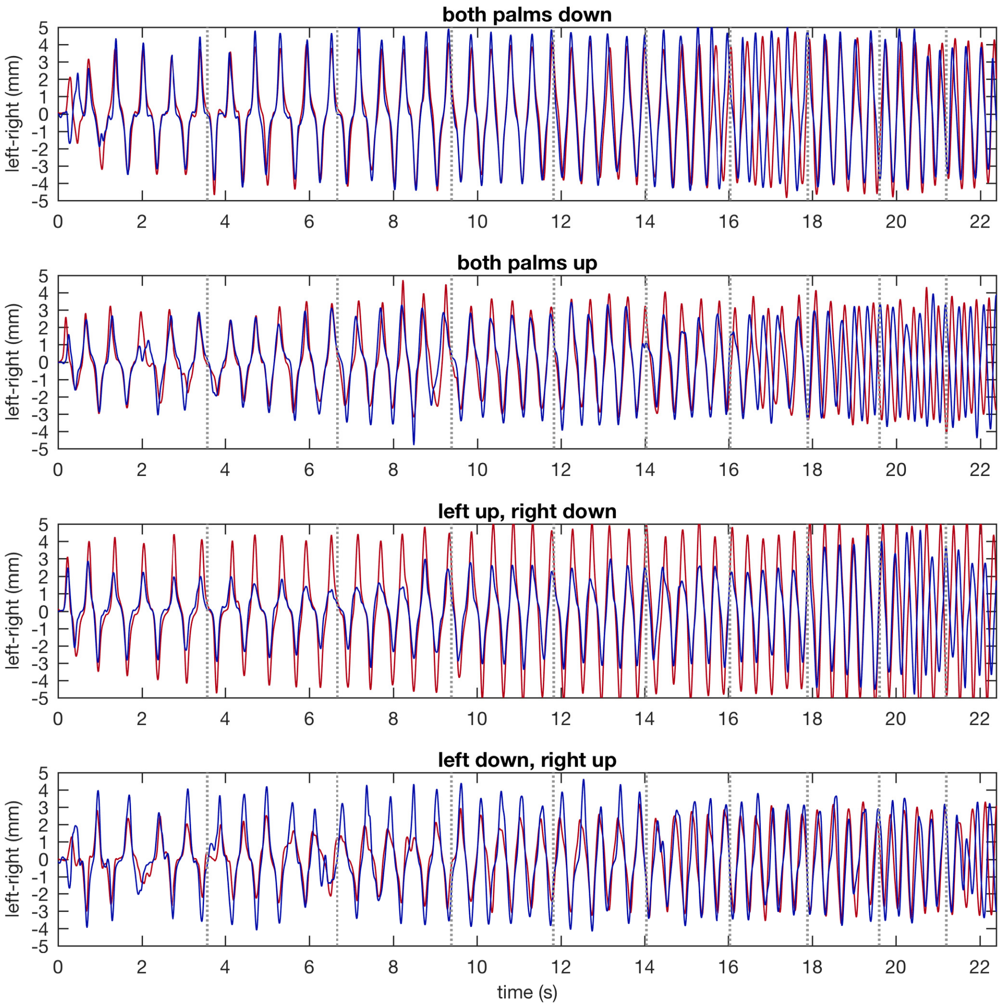

Figure S11: Left-right (horizontal) velocity traces of a single participant across hand orientations. Data are from the most difficult experimental condition, that is, parallel movements executed while receiving mirrored visual feedback. See Figure S12 for the corresponding traces in the up-down (vertical) dimension.

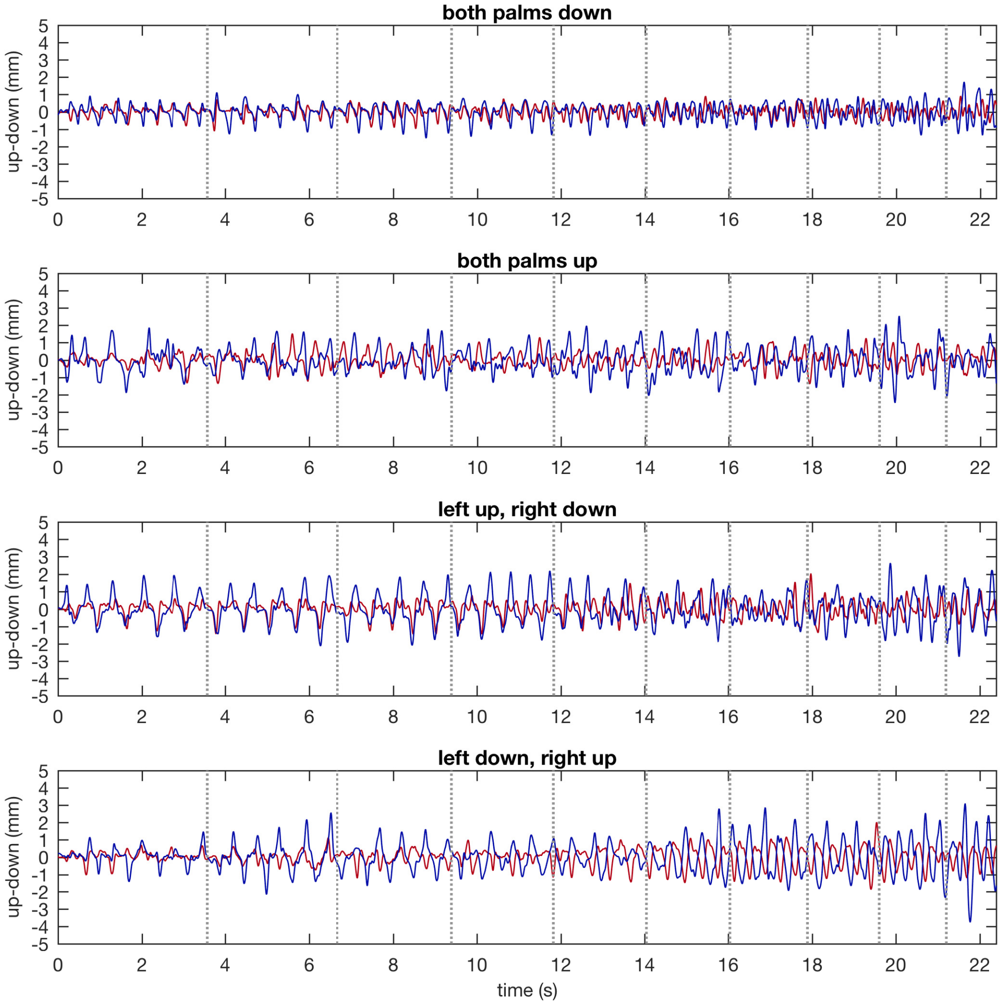

Figure S12: Up-down (vertical) velocity traces of the same participant and conditions as Figure S11.

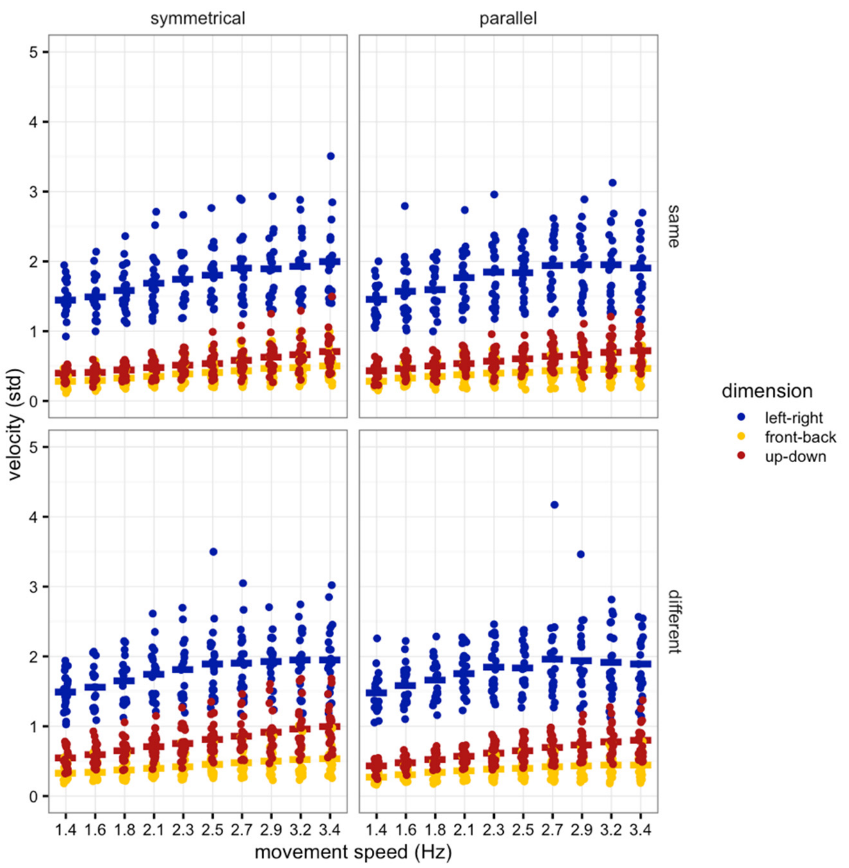

Figure S13: Illustration of the standard deviation of velocity per participant and speed level (1.4 to 3.4 Hz) for all three movement dimensions, averaged across trials and hands. The grand mean of standard deviations across participants is displayed as horizontal bars color-coded according to movement dimension.

Note, that a switch to vertical instead of horizontal movement patterns does not appear to have been an issue, as demonstrated above. However, some transitions from parallel to symmetrical coordination patterns occurred at high movement frequencies. This is a well-described phenomenon shown by numerous previous investigations [e.g., 6–8]. In the present study, these transitions were evident in an increased number of movements that were generated with relative phase differences centered around 180° when a phase difference around 0° characterizing parallel movements would have been appropriate (see Figure 1 in the manuscript for illustration of observed phase differences). Crucially, the question central to the present study was to test whether visual reafferent information is relevant for bimanual coordination. Our approach is to test whether the percentage of correctly performed movements decreases with speed. It is not essential for the current research question to further qualify whether or not pattern transitions occurred. Consequently, we merely dichotomized relative phases into correct and false responses instead of analyzing actual phase differences (see above, comparability with previous studies).

References

1. Sedlacek, M. & Krumpholc, M. Digital measurement of phase difference - a comparative study of DSP algorithms. *Metrol. Meas. Syst.* **Vol. 12,** 427–448 (2005).
2. Sedláček, M. Digital measurement of phase difference of LF signals—a comparison of DSP algorithms. in *Proceedings of IMEKO XVII World Congress (CD), Dubrovnik, Croatia* 639–644 (2003).
3. Wilson, A. D., Snapp-Childs, W. & Bingham, G. P. Perceptual learning immediately yields new stable motor coordination. *J. Exp. Psychol. Hum. Percept. Perform.* **36,** 1508 (2010).
4. Mechsner, F., Kerzel, D., Knoblich, G. & Prinz, W. Perceptual basis of bimanual coordination. *Nature* **414,** 69–73 (2001).
5. Heed, T. & Röder, B. Motor coordination uses external spatial coordinates independent of developmental vision. *Cognition* **132,** 1–15 (2014).
6. Cohen, L. Synchronous bimanual movements performed by homologous and non-homologous muscles. *Percept. Mot. Skills* **32,** 639–644 (1971).
7. Kelso, J. A. S. Phase transitions and critical behavior in human bimanual coordination. *Am. J. Physiol. - Regul. Integr. Comp. Physiol.* **246,** R1000–R1004 (1984).
8. Kelso, J. A. S., Scholz, J. P. & Schöner, G. Nonequilibrium phase transitions in coordinated biological motion: critical fluctuations. *Phys. Lett. A* **118,** 279–284 (1986).
